# Dietary Replacement Impacts of Fish Meal With Corn Gluten Meal on the Growth, Feed Utilization, and Biochemical Composition of Juvenile Red Sea Bream (*Pagrus major*)

**DOI:** 10.1155/anu/8361723

**Published:** 2025-05-23

**Authors:** Tae Woong Kwon, Sung Hwoan Cho

**Affiliations:** ^1^Ocean Science and Technology School, National Maritime and Ocean University, Busan 49112, Republic of Korea; ^2^Division of Convergence on Marine Science, National Korea Maritime and Ocean University, Busan 49112, Republic of Korea

**Keywords:** corn gluten meal, fish meal replacement impact, *Pagrus major*, regression analysis

## Abstract

The reliance on fish meal (FM) as the primary protein source in aquafeeds is becoming increasingly unsustainable due to overfishing and limited resources. Therefore, finding an alternative protein source for FM is a crucial issue in the research of aquafeed nutrition. Corn gluten meal (CGM) is a plant protein source commonly used in aquafeeds that has been considered a practical substitute for FM. This study is designed to evaluate the impacts of substituting FM with CGM in the feed of red sea bream (*Pagrus major*) on growth, feed utilization, and biochemical composition. Total of 600 juvenile (8.60 ± 0.011 g; initial mean body weight ± SE) fish were assigned to twelve 300 L flow-through tanks. The control (Con) diet included 55% FM. In the Con diet, 20%, 40%, and 60% of FM were replaced with CGM, identified as the CG20, CG40, and CG60 diets, respectively. Four isoproteic (51.5%) and isolipidic (14.5%) diets were prepared. All diets were supplied to triplicate groups of fish. Fish were hand-fed to satiation level twice daily for 56 days. At the completion of the 56-day feeding trial, the weight gain (WG) and specific growth rate (SGR) of red sea bream fed the Con and CG20 diets were superior (*p* < 0.0001 for both) to fish fed the CG40 and CG60 diets. WG and SGR of red sea bream linearly decreased with elevated dietary FM replacement levels (*Y* = −5.003333*X* + 38.9667, *R*^2^ = 0.9004, *p* < 0.0001 and *Y* = −0.002607*X* + 0.0314, *R*^2^ = 0.9083, *p* < 0.0001, respectively). Feed consumption (FC), protein efficiency ratio (PER), and protein retention (PR) of red sea bream fed the Con and CG20 diets were statistically (*p* < 0.006, *p* < 0.003, and *p* < 0.004, respectively) higher than those of fish fed the CG40 and CG60 diets. The feed conversion ratio (FCR) of red sea bream fed the CG40 and CG60 diets were statistically (*p* < 0.0002) higher than that of fish fed the Con and CG20 diets. The hepatosomatic index (HSI) of fish fed the Con and CG20 diets was statistically (*p* < 0.005) lower than that of fish fed the CG40 and CG60 diets. Neither the plasma and serum parameters nor the biochemical composition except for arginine and lysine content of the whole-body fish were statistically (*p* > 0.05) altered by dietary FM substitution with CGM. In conclusion, FM up to 20% could be replaced by CGM in the diet of red sea bream without bringing about negative impacts on the growth, feed utilization, biological indices (except for HSI), blood chemistry, proximate composition, amino acid (AA; except for arginine and lysine content), and fatty acid (FA) profiles.

## 1. Introduction

Fish meal (FM) is extensively used as the primary protein source in fish feeds because it is rich in essential nutrients, such as proteins including nondispensable essential amino acid (EAA) and lipids including nondispensable fatty acids (FAs) and has high digestibility [1, 2]. Generally, FM is an important ingredient in the feeds of carnivorous fish species, since they require diets with higher protein content than omnivorous fish or herbivorous fish [3]. Nevertheless, global FM production has exhibited consistently unstable fluctuations from 2002 to 2022, peaking at 5578 thousand metric tons (MT) in 2003 and reaching its lowest value at 4244 thousand MT in 2009 [4]. Overfishing and abrupt climate change might influence these fluctuations in global FM production [5, 6]. Additionally, FM demand has also increased from the growing global aquaculture industry globally also rises [7] and influences FM market prices [8]. Therefore, to ensure sustainable fish farming, feed nutritionists should try to identify a suitable substitute for FM in aquafeeds [9].

Stable year-round production, high productivity, cost effectiveness, and balanced nutrition for fish should be considered to look for a replacer for FM in fish feeds [1, 10]. Plant-based alternative protein sources have advantages over animal-based proteins, such as the absence of zoonotic diseases for fish and more inexpensive market prices than FM [9]. Numerous studies on alternative plant-based protein sources for FM in fish diets, such as corn gluten meal (CGM), soybean protein concentrate, fermented rapeseed meal, and corn protein concentrate (CPC) [1, 11–13] have been carried out.

CGM is a by-product of processing corn and one of the most commonly used plant protein sources for FM substitute in aquafeeds [10]. It is recognized as a high protein, low fiber, and vitamin-rich feed ingredient that provides sufficient EAA content except for arginine and lysine [1, 10, 14]. Specifically, CGM has been considered as a good substitute for FM in the diets of spotted rose snapper (*Lutjanus guttatus*), Asian seabass (*Lates calcarifer*), puffer (*Takifugu fasciatus*), olive flounder (*Paralichthys olivaceus*), and rockfish (*Sebastes schlegeli*) [1, 10, 14–16].

Red sea bream is a carnivorous species and highly required for FM, innovation and research can help it get better [17]. In the Republic of Korea (henceforth, Korea), red sea bream is one of the extensively cultured marine finfish species, with aquaculture production rising from 4520 MT in 2006 to 8078 MT in 2022, representing an increase of approximately 1.79 times over this period [18].

A variety of plant protein source has been developed for substitution of FM in the feeds of red sea bream [10, 11, 19–21]. In particular, 50% of FM could be replaced with the combined meat meal (MM) and chicken by-product meal (CBM) in a 60% FM-based diet without significantly lowering weight gain (WG) of red sea bream when juvenile fish were provided with a 60% FM (30% tuna by-product meal and 15% each pollock and sardine FM)–based diet or one of diets substituting 50% of FM with various plant protein sources (CGM and soy protein concentrate (SPC)), animal protein sources (CBM and MM), or their combinations [22]. This prior research also suggested that the substitutability of CGM for FM with a lower level than 30% should be tested in the diet of red sea bream. In the study on red sea bream [23], replacing up to 30% and 70% of FM in the 50% FM–based diets with CGM in the diets did not compromise the growth of fingering fish, which grew from 53 to 89 g, and grower fish, which grew from 280 to 751 g, respectively, when both fish were fed with a 50% FM–based diet or one of diets replacing 30%, 50%, 70%, 90%, and 100% of FM with CGM for periods of 40–232 days, respectively. Furthermore, the substitutability of fermented soybean, MM, and tuna by-product meal or FM in diets varied significantly depending on the size (age) of fish [16, 24–26].

This experiment, thus, aimed to estimate the substitution impacts of various levels of FM with CGM on growth, feed utilization, and biochemical composition of juvenile red sea bream.

## 2. Materials and Methods

### 2.1. Fish Rearing Conditions

The experimental fish were purchased from a private hatchery (Tongyeong–si, Gyeongsanggnam–do, Korea). Fish were supplied with a commercially extruded pellet containing 50% crude protein and 13% crude lipid (Suhyup Feed, Uiryeong–gun, Gyeongsangnam–do, Korea) twice daily at a ratio of 2%–3% biomass for a 2-week acclimatization period of the experiment at the Institute of Ocean Science Education, Gangneung–Wonju National University (Gangneung-si, Gangwon-do, Korea). After this period, a total of 600 juvenile (8.60 ± 0.011 g; initial mean body weight ± SE) fish was randomly placed in twelve 300 L flow-through tanks (water volume: 260 L; 50 fish/tank). A blend of underground seawater and sand-filtered seawater at a 1:1 was provided to each tank at a flow rate of 4.4 L/min throughout the feeding experiment. Proper aeration was provided to each tank.

Water quality was monitored daily using a water quality meter (AZ–8603; AZ Instrument Corp., Taichung, Taiwan) throughout the 56-day feeding experiment as follows: water temperature ranged from 17.2 to 23.1°C (20.6 ± 1.54°C; mean ± SD), dissolved oxygen ranged from 7.4 to 8.3 mg/L (7.7 ± 0.27 mg/L), salinity ranged from 29.7 to 31.5 g/L (30.5 ± 0.40 g/L), and pH ranged from 7.4 to 7.7 (7.5 ± 0.07). The photoperiod coincided with natural cycles. The bottoms of each tank were siphon-cleaned daily. Dead fish were removed upon observation and weighed.

### 2.2. Preparation of the Experimental Diets

Four experimental isoproteic (51.5%) and isolipidic (14.5%) feeds were prepared (Table 1). All experimental diets were formulated to satisfy the dietary protein and lipid needs of red sea bream [27]. The protein sources were 55% anchovy meal as FM source and 17% fermented soybean meal in the control (Con) diet. Additionally, in the Con diet, 17.5% wheat flour and 4% of both fish and soybean oils were included as the carbohydrate and lipid sources, respectively. In the Con diet, 20%, 40%, and 60% of FM were replaced with CGM, identified as the CG20, CG40, and CG60 diets, respectively. All experimental feeds were supplied to triplicate groups of fish.

The ingredients of the experimental feeds were uniformly blended with water at a ratio of 3:1 and then, pelletized in a 3 mm die using a pelletizing machine (SMC-32; SL Company, Incheon, Korea). The experimental feeds were dried at 40°C in an electronic drier (Jinwoo Electronics Co., Ltd., Hwaseong–si, Gyeonggi–do, Korea) for 48 h and kept in a freezer at −20°C until used. Red sea bream were hand-fed to visual satiation twice (08:30 and 17:30) daily for 8 weeks.

### 2.3. Assessment of Biological Indices of Red Sea Bream

Following the 56-day feeding period, all live fish were starved for 24 h and then, anesthetized with tricaine methanesulfonate (MS-222) at 100 mg/L. The number of surviving fish from each tank was counted and collectively weighed to calculate survival and WG. The biological indices of 10 anesthetized fish from each tank were measured. The formula for calculating growth performance, feed utilization, and biological indicators (condition factor (*K*), hepatosomatic index (HSI), and viscerosomatic index (VSI)) were the same as in Baek and Cho's [28] study.

### 2.4. Analysis of Blood Chemistry of Fish

At the completion of the feeding trial, blood samples were collected using a 1 mL heparinized syringe from the caudal vein of five anesthetized fish randomly selected from each tank. After centrifugation at 2716 × *g* at 4°C for 10 min, plasma samples were collected and immediately preserved in a freezer at −70°C for analysis of alanine aminotransferase (ALT), aspartate aminotransferase (AST), alkaline phosphatase (ALP), total bilirubin (T-BIL), total cholesterol (T-CHO), triglyceride (TG), total protein (TP), and albumin (ALB) using an automated chemistry system (Fuji Dri-Chem NX500i; Fujifilm, Tokyo, Japan).

A 1 mL syringe was used to collect blood samples from the caudal vein of five randomly chosen anesthetized fish from each tank. Then the serum samples were separated by centrifugation at 2716 × *g* at 4°C for 10 min and immediately preserved in a freezer at −70°C until analysis. The same protocols and methods employed by Lee et al.'s [29] study for the analysis of superoxide dismutase (SOD) and lysozyme activity for rockfish were utilized in this study.

### 2.5. Biochemical Composition of the Experimental Feeds and Fish

All fish from each tank that survived beyond the 56-day feeding experiment (≥20 fish), 10 fish from before the experiment, and the experimental diets were homogenized to analyze their chemical compositions. The chemical compositions of the whole-body fish and experimental feeds were confirmed using the AOAC standard method [30]. The moisture content of the experimental feeds and the whole-body fish were determined using a dry oven at 105°C for 6 h and 24 h, respectively. The ash content was determined using a muffler at 550°C for 4 h. The semiauto Kjeldahl method (Kjeltec 2100 Distillation Unit; Foss, Hillerød, Denmark) was used to determine the crude protein content and the crude lipid content was determined using the ether extraction method (Soxtec 2043 Fat Extraction System; Foss, Hillerød, Denmark). The gross energy content of the experimental diets was evaluated using an Isoperibol Calorimeter 6100EF calorimeter (Parr Instrument Co., Moline, IL, USA) equipped with an oxygen bomb model 1108, to measure the heat generated upon complete combustion of the experimental diets.

The ninhydrin postcolumn reaction method was used for analysis with ion-exchange chromatography. The amino acids (AAs) except for tryptophan in the experimental feeds and whole-body fish were analyzed using an AA analyzer (L-8800 Auto-analyzer; Hitachi Ltd., Tokyo, Japan). For the AA analysis, 0.2 g of the sample was placed in a decomposition tube. Then, 30 mL of 6 N HCl was added. Nitrogen gas was then injected and the mixture was hydrolyzed at 110°C for 24 h. The filtrate was concentrated using a reduced pressure concentrator, filtered using a 0.45 μm nylon syringe filter, and then, used for analysis. Additionally, tryptophan content of the samples was determined using high-performance liquid chromatography (S1125 HPLC pump system; Sykam GmbH, Eresing, Germany).

The experimental diets and whole body of fish were compared to known standards (37 component FAME mix CRM47885; Supelco, St. Louis, MO, USA) to identify FA. Lipids for the FA analysis from the samples were extracted using a mixture of chloroform and methanol at a 2:1 ratio, as per the Folch et al.'s [31] method. Gas chromatography (HP 6890; Hewlett Packard Inc., Palo Alto, CA, USA) equipped with an SP-2560 capillary column (inner diameter 100 m × 0.25 mm and film thickness 0.20 μm; Supelco, Bellefonte, PA, USA) and a flame ionization detector was used to analyze the FA methyl esters, which were prepared through trans-esterification using a 14% BF_3_-MeOH (Sigma, St Louis, MO, USA) solution.

### 2.6. Statistical Analysis

One-way analysis of variance (ANOVA) and Duncan's multiple range test [32] were used to determine significant (*p* < 0.05) differences of mean using IBM SPSS Statistics for Windows, Version 24.0 (IBM Corp., Armonk, NY, USA). All data were assessed for normality and homogeneity by Shapiro–Wilk's and Levene's tests, respectively, prior to analysis. Percentage data was arcsine-transformed prior to statistical analysis. The data with significant differences were subjected to regression analysis to determine the optimal model. Additionally, orthogonal polynomial contrast was conducted to determine whether all parameters or dietary CGM replacement levels were exhibited as linear, quadratic, and cubic.

## 3. Results

### 3.1. Amino and FA Profiles of the Main Proteins and Experimental Diets

CGM had comparatively high leucine and phenylalanine content among EAA over FM (Table 2). As the dietary FM substitution with CGM increased, leucine and phenylalanine content also increased. The Con and CG20 diets appeared to fulfill the red sea bream's dietary arginine requirement, but the CG40 and CG60 diets appeared to provide less than their dietary requirement [33]. All experimental diets met the red sea bream's lysine and valine dietary requirements [34, 35].

The total saturated FA (*∑*SFA), total monounsaturated FA (*∑*MUFA), and total *n*−3 highly unsaturated FA (*∑n*−3 HUFA) including eicosapentaenoic acid (EPA, C20:5*n*−3) and docosahexaenoic acid (DHA, C22:6*n*−3) in FM were comparatively high over those in CGM (Table 3). Elevated dietary FM replacement by CGM resulted in decreased *∑*SFA, *∑*MUFA, and *∑n*−3 HUFA.

### 3.2. Red Sea Bream Survival and Growth

**Figure 1:**
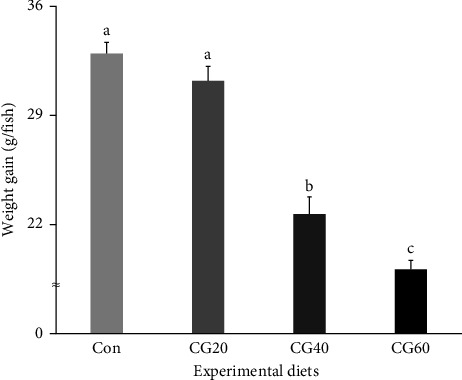
Weight gain (WG, g/fish) of red sea bream (*Pagrus major*) fed experimental diets substituting various levels of fish meal with corn gluten meal (means of triplicate ± SE; *p* < 0.0001). (Regression analysis: *Y* = −5.003333*X* + 38.9667, *R*^2^ = 0.9004, *p* < 0.0001; orthogonal polynomial contrast: linear = 0.0001, quadratic = 0.3428, and cubic = 0.3428).

**Figure 2:**
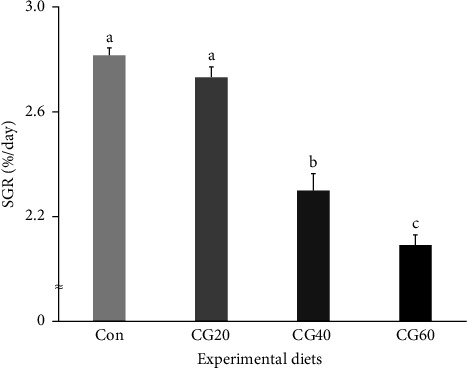
Specific growth rate (SGR, %/day) of red sea bream (*Pagrus major*) fed the experimental diets substituting various levels of fish meal with corn gluten meal (means of triplicate ± SE; *p* < 0.0001). (Regression analysis: *Y* = – 0.002607*X* + 0.0314, *R*^2^ = 0.9083, *p* < 0.0001; orthogonal polynomial contrast: linear = 0.0001, quadratic = 0.1741, and cubic 0.0161).

Survival of fish ranged from 86.0% to 91.3% and was not statistically (*p* > 0.5) influenced by dietary FM replacement with CGM (Table 4). WG (g/fish) and specific growth rate (SGR, %/day) of fish supplied with the Con and CG20 diets were statistically (*p* < 0.0001 for all) superior compared to fish supplied with the CG40 and CG60 diets (Figures 1 and 2, respectively). In orthogonal polynomial contrast, WG and SGR of fish revealed linear (*p*=0.0001 for both) and cubic (*p*=0.0161 for SGR) relationships with dietary FM substitution levels with CGM. In regression analysis, linear relationships were found to be the most fitting models between dietary replacement levels of FM with CGM (*X*) and WG (Figure 1; *Y* = −5.003333*X* + 38.9667, *R*^2^ = 0.9004, *p* < 0.0001) and SGR (Figure 2; *Y* = −0.002607*X* + 0.0314, *R*^2^ = 0.9083, *p* < 0.0001).

### 3.3. Red Sea Bream Feed Consumption (FC) and Feed Utilization

**Figure 3:**
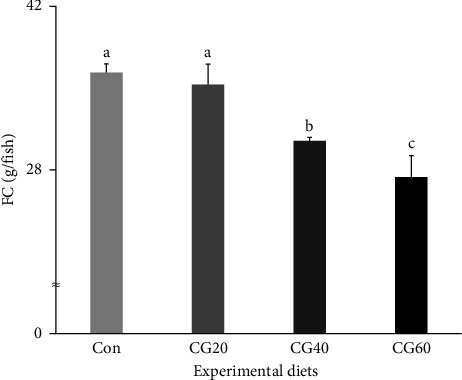
Feed consumption (FC, g/fish) of red sea bream (*Pagrus major*) fed the experimental diets substituting various levels of fish meal with corn gluten meal (means of triplicate ± SE; *p* < 0.006). (Regression analysis: *Y* = – 3.161333*X* + 40.2500, *R*^2^ = 0.7418, *p* < 0.0004; orthogonal polynomial contrast: linear = 0.0008, quadratic = 0.4652, and cubic = 0.3946).

The FC, protein efficiency ratio (PER), and protein retention (PR) of fish supplied with the Con and CG20 diets were statistically (*p* < 0.006, *p* < 0.003, and *p* < 0.004, respectively) greater than those of fish supplied with the CG40 and CG60 diets (Figure 3 and Table 4). The feed conversion ratio (FCR) of fish supplied with the CG40 and CG60 diets were statistically (*p* < 0.0002) higher than that of fish supplied with the Con and CG20 diets. In orthogonal polynomial contrast, the FC, FCR, PER, and PR revealed linear (*p*=0.0008, *p*=0.0001, *p*=0.0005, and *p*=0.0006, respectively) relationships with dietary substitution levels of FM with CGM. In the regression analysis, linear relationships were identified as the most fit model between dietary replacement levels of FM with CGM (*X*) and FC (*Y* = – 3.161333*X* + 40.2500, *R*^2^ = 0.7418, *p* < 0.0004), FCR (*Y* = 0.127333*X* + 0.8767, *R*^2^ = 0.8621, *p* < 0.0001), PER (*Y* = −0.149000*X* + 1.9517, *R*^2^ = 0.7435, *p* < 0.0004), and PR (*Y* = − 0.023857*X* + 0.3166, *R*^2^ = 0.7208, *p* < 0.0006).

### 3.4. Red Sea Bream Biological Indices

The *K* of fish changed from 1.85 to 1.91 and VSI changed from 8.76% to 9.58%. *K* and VSI of fish were not statistically (*p* > 0.2 for both) affected by dietary FM replacement with CGM. However, HSI of fish fed the CG40 and CG60 diets were statistically (*p* < 0.005) greater than that of fish fed the Con and CG20 diets. In orthogonal polynomial contrast, a linear (*p*=0.0011) relationship was found in the HSI of fish with increasing dietary FM substitution with CGM. In the regression analysis, a linear relationship was found to be the most suitable model between dietary FM replacement by CGM (*X*) and HSI (*Y* = 0.001237*X* + 0.0257, *R*^2^ = 0.6583, *p* < 0.002).

### 3.5. Red Sea Bream Blood Chemistry

The red sea bream's plasma AST ranged from 48.44 to 53.33 U/L, ALT ranged from 8.67 to 9.11 U/L, ALP ranged from 168.89 to 174.67 U/L, T-BIL ranged from 0.80 to 0.92 mg/dL, T-CHO ranged from 248.56 to 256.00 mg/dL, TG ranged from 405.11 to 419.11 mg/dL, TP ranged from 4.68 to 5.02 g/dL, and ALB ranged from 0.97 to 1.08 g/dL (Table 5). These plasma parameters were not statistically (*p* > 0.05 for all) influenced by dietary FM substitution with CGM.

Furthermore, serum SOD varied from 3.91 to 4.53 ng/mL and lysozyme activity varied from 34.93 to 48.51 U/mL. None of the serum parameters were statistically (*p* > 0.9 and *p* > 0.5, respectively) influenced by dietary FM replacement with CGM.

### 3.6. Biochemical Composition of the Whole-Body Red Sea Bream

The whole-body red sea bream moisture content ranged from 69.27% to 69.40%, crude protein content ranged from 16.07% to 16.24%, crude lipid content ranged from 8.70% to 8.98%, and ash content ranged from 4.32% to 4.63% (Table 6). The whole-body fish chemical composition was not statistically (*p* > 0.9, *p* > 0.9, *p* > 0.8, and *p* > 0.6, respectively) influenced by dietary FM replacement by CGM.

The AA profiles of the whole-body red sea bream, except for arginine and lysine, were not statistically (*p* > 0.05 for all) influenced by dietary FM replacement by CGM (Table 7). The arginine and lysine content of fish supplied with the Con and CG20 diets were statistically (*p* < 0.0006 and *p* < 0.002, respectively) greater than those of fish supplied with the CG40 and CG60 diets. In orthogonal polynomial contrast, the arginine and lysine of the whole-body fish revealed linear (*p*=0.0001 and *p*=0.0002, respectively) relationships with dietary FM substitution levels associated with CGM. In the regression analysis of the whole-body fish, linear relationships were detected to be the best suitable model between dietary FM substitution with CGM (*X*) and arginine (*Y* = −0.096333*X* + 1.1033, *R*^2^ = 0.8602, *p* < 0.0005) and lysine (*Y* = −0.075333*X* + 1.3383, *R*^2^ = 0.8233, *p* < 0.0001) of the whole-body fish.

Last, the FA profiles of the whole-body red sea bream were not statistically (*p* > 0.05 for all) influenced by dietary FM substitution by CGM (Table 8).

## 4. Discussion

The SGR values (2.09–2.81%/day) of fish (initial body weight of 8.6 g) in the current study were comparable to those reported (2.37–2.62, 1.74–2.10, and 2.49–2.96%/day) in the same fish species of similar size (initial body weight of 4.5, 8.5, and 5.9 g, respectively) [12, 21, 36], demonstrating that growth performance of red sea bream was reasonably attained in the current study. Furthermore, no statistical differences in WG and SGR of fish fed the Con and CG20 diets were observed, but they were superior compared to fish fed the CG40 and CG60 diets, also indicating that CGM could be substituted up to 20% of FM in the 55% FM–based diet of red sea bream without lowering WG and SGR. However, Takagi et al. [23] demonstrated that FM up to 30% and 70% could be replaced by CGM in the diets of red sea bream without compromising growth, FCR, and PER in fingerling and grower red sea bream. Differences in substitutability of FM with CGM in the red sea bream diets appeared to be more associated with fish size (age): juvenile fish (initial mean body weight of 8.6 g) in this experiment vs. fingerling (initial mean body weight of 53.2 g) and grower fish (initial mean body weight of 280.0 g) in Takagi et al.'s [23] study. Smaller fish are more likely than larger fish to replace a smaller amount of FM with alternative protein sources in their diet [16, 24–26, 37]. For instance, Lee et al. [16] unveiled that 6% and 24% FM protein could be substituted by fermented soybean meal in diets for juvenile and grower rockfish, respectively, when they were supplied with a 58% FM–based feed or feeds substituting 6%, 12%, 18%, and 24% FM protein with fermented soybean meal for 8 weeks.

In the current experiment, WG and SGR of fish were found to linearly decrease with elevated levels of dietary FM replacement with CGM based on regression analysis. Furthermore, a linear model (for WG and SGR) and a cubic model (for SGR) were determined to be the most appropriate model between the growth performance (WG and SGR) of fish and the levels of dietary FM replacement with CGM, as assessed through orthogonal polynomial contrast. Similarly, FM up to 14.8% (8% FM protein in the diet) could be substituted with CGM without compromising growth, feed intake, and digestibility of puffer when juvenile puffer were provided with a 54% FM–based diet or diets substituting 7.4%, 14.8%, 22.2%, and 29.6% FM with CGM [14]. Likewise, Nandakumar et al. [10] explained that CGM can be a potential feed ingredient in the diet of Asian seabass and can replace FM up to 28.6% without negatively impacting on growth and digestibility when fingerling Asian seabass were supplied with a 35% FM–based diet or diets replacing 14.3%, 28.6%, 42.9%, and 57.1% of FM with CGM.

The dietary lysine and valine requirements of red sea bream were met in all experimental diets in this study [34, 35]. However, their arginine requirements [33] were fulfilled in the Con and CG20 diets, but not in the CG40 and CG60 diets. This could be why growth performance of fish fed the Con and CG20 diets was superior to fish fed the CG40 and CG60 diets. Arginine is one of the EAA affecting fish growth and is likely deficient in CGM [38, 39]. For instance, Hernández et al. [1] showed that up to 60% of FM (32.9% FM protein) in a diet could be replaced with CGM without lowering growth, FC, FCR, and PER of spotted rose snapper when juveniles were provided with a 54.8% FM–based diet or diets replacing 20% and 40% of FM with CGM supplemented with arginine only and 60%, 80%, and 100% of FM with CGM supplemented with both arginine and lysine.

Substitutability of CGM for FM in diets of commercially important marine fish is given in Table 9. Substitutability of CGM for FM up to 20% in the diet of red sea bream in this experiment is similar (29% and 30%) to that in the diets of some marine fish species [10, 23], but lower and higher than that in the diet of Puffer (*Takifugu fasciatus*) [14] and gilthead sea bream (*Sparus aurata*) [41]. Furthermore, substitutability of FM with CGM for the smaller red sea bream in this study (20%) appeared to be lower than for the larger fish (30% and 70% for juvenile and grower red sea bream grown from 53 to 89 g and 280 to 751 g, respectively) [23]. Moreover, substitutability of FM with CGM supplemented EAA, which are likely to be lack or deficient in CGM, in diets of marine fish seemed to be improved (40%–60%) [1, 15, 41]. Thus, it was suggested that substitutability of CGM for FM in diets varies highly depending on fish species, fish size (age), and supplementation of EAA.

The *∑n*−3 HUFA and the ratio of DHA to EPA tended to decrease linearly with increased FM replacement with CGM in the experimental diets in this study. This might have negatively affected growth of red sea bream. For example, WG and daily growth index of golden gray mullet (*Liza aurata*) fed a diet containing 0.2% *∑n*−3 HUFA were poorer than those of of fish fed a diet containing 1.2% *∑n*−3 HUFA when fish were fed diets containing 0.2% and 1.2% *∑n*−3 HUFA [42]. In gilthead seabream, WG and daily growth index decreased linearly as dietary ratio of DHA to EPA decreased, with diets containing DHA to EPA ratios ranging from 1.11 to 1.45 [43]. Although dietary optimal ratio of DHA to EPA varied depending on fish species [44], it remains unknown for red sea bream.

In this study, the FC of red sea bream supplied with the Con and CG20 diets were higher than that of fish supplied with the CG40 and CG60 diets in the current experiment. This also indicated that up to 20% of FM in the 55% FM–based diet could be substituted with CGM without reducing FC. Nevertheless, the FC of fish linearly lowered with elevating levels of CGM substitution for FM in diets based on regression analysis. Additionally, regarding orthogonal polynomial contrast, a linear model was the most appropriate between FC and FM replacement levels with CGM in the diets. Decreased WG and SGR of red sea bream were well reflected from reduced FC with elevated dietary FM replacement with CGM in this study. Similarly, elevated FM substitution levels with plant protein sources in diets commonly led to reduced FC of fish because of reduced palatability and eventually lowered growth performance. For instance, elevated levels of FM replacement with CGM in diets have been associated with reduced palatability and decreased FC and growth performance in seabass and puffer [14, 45]. Plant proteins contain fewer palatable substances, commonly leading to less palatable plant protein-rich diets [46, 47]. Arginine, lysine, and glycine are commonly recognized as feed stimulants and/or enhancers in aquatic animals [48] and elevated FM substitution with CGM in diets resulted in decreased arginine, lysine, and glycine content in this experiment, leading to FC reduction in red sea bream.

Superior FCR, PER, and PR were found in fish supplied with the Con and CG20 diets compared to fish supplied with the CG40 and CG60 diets and were attributed to lower arginine and lysine content in the latter diets. Likewise, elevated FM replacement by CGM in diets reduced the digestibility and availability of specific AA (arginine and lysine) and eventually lowered growth, FCR, and PER of turbot (*Psetta maxima*) in a prior study [49]. Bu et al. [50] also emphasized that greater (>40%) substitution of FM with CGM in feeds led to decreased WG, SGR, feed intake, and PER in *Pseudobagrus ussuriensis* because of their low arginine and lysine content when juvenile fish were supplied with a 50% FM–based diet or diets substituting 10%, 20%, 30%, 40%, 50%, and 60% of FM with CGM. Additionally, they estimated the suitable dietary substitution level to be 37.7% of FM with CGM in diet based on broken-line model analysis. Similarly, the poorest growth and lowest feed efficiency ratio were found in gilthead sea bream fed a diet substituting 80% of FM protein with CGM. This is because it had the lowest arginine and lysine content when juvenile fish were fed with a 61.5% FM–based diet or one of diets replacing 20%, 40%, 60%, and 80% of FM protein with CGM [41]. However, incorporated EAA that are likely to be deficient in a specific alternative ingredient could improve its substitutability for FM in diets. For instance, FM replacement up to 60% could be made in diet without compromising WG, SGR, feed intake, FCR, and PER of spotted rose snapper when juvenile fish were provided a 54.8% FM–basal diet or one of diets replacing 20%, 40%, 60%, 80%, and 100% of FM with CGM supplemented with arginine and lysine [1]. Men et al. [45] also reported that dietary 60% of FM with CGM could be made without reducing the growth performance of seabass when fish were supplied with a 52% FM–based diet or one of diets replacing 15%, 30%, 45%, 60%, and 75% of FM with CGM supplemented with arginine and lysine.

*K* is one of the most universally used morphometric indicators [51] and provides a traditional method to estimate fish body condition [52]. Additionally, VSI and HSI are reliable parameters for accessing fish health and nutrient utilization in feed [53]. In this experiment, the *K* and VSI of fish were unaffected by dietary FM substitution with CGM in the current study. Likewise, FM substitution with CGM in diets unaffected *K* and VSI of marine fish in prior research [1, 10, 14]. However, in the current experiment, higher HSI was found in fish supplied with the CG40 and CG60 diets compared with fish supplied with the Con and CG20 diets. Furthermore, according to regression analysis, the HSI of red sea bream linearly increased with dietary increased FM replacement by CGM. Increased HSI of fish could have resulted from higher carbohydrate content in the CG40 (29.4% carbohydrate) and CG60 (30.9% carbohydrate) compared to the Con (27.6% carbohydrate) and CG20 (28.2% carbohydrate) diets. High carbohydrate intake can also result in excessive glycogen deposition in the liver and enlarged it, potentially impairing liver function [54]. For example, the HSI of hybrid grouper (*Epinephelus lanceolatus* ♂ × *Epinephelus fuscoguttatus* ♀) and olive flounder tended to increase with elevated levels of dietary FM substitution with the combination of soybean meal, CGM and cottonseed meal, and soybean meal, respectively [55, 56]. Furthermore, when the levels of TG exceed the adaptative capacity of the body, TG accumulation could occur in hepatocytes [57]. The HSI of large yellow croaker (*Larimichthys crocea*) fish fed a higher lipid diet increased because of accumulation of hepatic TG when fish were fed diets containing 12% and 18% lipid content [58]. Hepatic metabolism and digestive efficiency in largemouth bass (*Micropterus salmoides*) were impaired when fish were fed a 24% FM–based diet deficient in arginine, contributing to elevated HSI and liver dysfunction [59]. Golden pompano (*Trachinotus ovatus*) fed a diet deficient in *n*−3 HUFA exhibited an increased HSI because of abnormal hepatic lipid metabolism and excessive accumulation of hepatic TG [60]. In cobia (*Rachycentron canadum*), increasing FM replacement levels with lupin kernel meals in diets linearly reduced nutrient digestibility, leading to the accumulation of hepatic lipid and increased HSI [61].

As animal blood without blood cells, plasma is a key biological liquid used for clinical diagnostics [62]. Here, the plasma measurements of fish were unaffected by dietary FM substitution by CGM in this experiment, indicating that 60% of FM could be substituted with CGM in diets without harming the plasma measurements of fish. Similarly, 50% of FM could be replaced with various plants (CGM and SPC), animals (MM and CBM), and their blends in a 60% FM–based feed without bringing about negative impacts on plasma measurements of red sea bream [22]. Additionally, plasma measurements of olive flounder were not altered by 25% and 50% of FM replacements with CGM, SPC, and CPC in diets supplemented with 12% jack mackerel meal [46]. However, Dossou et al. [12] showed that glucose, AST, and ALT of red sea bream were unaffected, but that cholesterol and TG were affected by substituting 50% of FM with rapeseed meal or fermented rapeseed meal.

SOD is the most powerful cellular antioxidant and represents the first line of antioxidant defense of an organism against biological oxidants [63]. Lysozyme is a cornerstone of innate immunity and important for the resolution of inflammation at mucosal sites [64]. Here, the serum SOD and lysozyme activity of fish were unaffected by dietary FM replacement by CGM, indicating that dietary FM replacement by CGM led to no undesirable impact on serum parameters. Similarly, lysozyme activity and SOD of olive flounder and rockfish unaffected by 25% and 50% of FM substitution by CGM, SPC, and CPC in diets [46, 47].

The lack of statistical differences in the proximate composition, the AA profiles except for arginine and lysine content, and the FA profiles of the whole-body red sea bream might imply that FM could be replaced with CGM in diets without deteriorating the proximate composition and FA profiles of the whole-body fish. However, in this experiment, decreased arginine and lysine content of whole-body fish were directly associated with their decreased content and increased FM substitution with CGM in diets in this experiment. Likewise, arginine and lysine content of the whole-body fish decreased with increased dietary FM replacement with CGM according to regression analysis. Similarly, Li et al. [65] demonstrated that lysine and methionine content of the whole-body of *Nibea diacanthus* were directly reflected from their dietary content when various levels of FM in diets were replaced with fermented soybean meal. Similarly, arginine content of the whole-body in gibel carp (*Carassius auratus gibelio*) and Mediterranean yellowtail (*Seriola dumerili*) were strongly associated with their dietary content when FM was replaced with alternative sources in diets [66, 67]. However, the chemical composition and AA and FA profiles of the whole-body of Senegalese sole (*Solea senegalensis*) were influenced by FM substitution with blended soybean meal, corn gluten, peas, and wheat meal in diets [68].

## 5. Conclusion

FM up to 20% could be substituted with CGM in a 55% FM–based diet without harming WG, SGR, FC, feed utilization, blood chemistry, and biochemical composition of red sea bream and only reducing arginine and lysine content. However, EAA supplementation, particularly arginine and lysine that are likely deficient, may allow for higher levels (>20%) of FM replacement with CGM in the feed of red sea bream.

## Figures and Tables

**Table 1:** Ingredient and chemical composition of the experimental diets (%, DM basis).

	Experimental diets			
	Con	CG20	CG40	CG60
Ingredients (%, DM)				
Fish meal^a^	55.0	44.0	33.0	22.0
Corn gluten meal (CGM)^b^	0.0	12.2	24.4	36.6
Fermented soybean meal	17.0	17.0	17.0	17.0
Wheat flour	17.5	15.1	12.7	10.3
Fish oil	4.0	5.2	6.4	7.6
Soybean oil	4.0	4.0	4.0	4.0
Vitamin premix^c^	1.0	1.0	1.0	1.0
Mineral premix^d^	1.0	1.0	1.0	1.0
Choline	0.5	0.5	0.5	0.5
Total	100.0	100.0	100.0	100.0
Nutrients (%, DM)				
Dry matter	95.7	95.6	96.1	95.3
Crude protein	51.3	51.5	51.4	51.7
Crude lipid	14.6	14.7	14.6	14.7
Ash	9.8	8.9	7.4	6.1
Gross energy (kJ/g)	18.1	18.2	18.5	18.7

^a^Fish meal (anchovy meal; crude protein: 73.4%, crude lipid: 10.7%, and ash: 14.0%) was imported from Chile (USD 1.84/kg FM, USD 1 = 1325 KRW).

^b^Corn gluten meal (CGM; crude protein: 69.3%, crude lipid: 0.6%, and ash: 1.0%) was purchased from Hyunjin Livestock Distribution Co., Ltd. (Incheon Metropolitan City, Korea; USD 0.80/kg).

^c^Vitamin premix (g/kg mix): L–ascorbic acid: 121.2; DL–*α*–tocopheryl acetate: 18.8; thiamin hydrochloride: 2.7; riboflavin: 9.1; pyridoxine hydrochloride: 1.8; niacin: 36.4; Ca–D–pantothenate: 12.7; myo–inositol: 181.8; D–biotin: 0.27; folic acid: 0.68; p–aminobenzoic acid: 18.2; menadione: 1.8; retinyl acetate: 0.73; cholecalciferol: 0.003; cyanocobalamin: 0.003.

^d^Mineral premix (g/kg mix): MgSO_4_·7H_2_O: 80.0; NaH_2_PO_4_·2H_2_O: 370.0; KCl: 130.0; ferric citrate: 40.0; ZnSO_4_·7H_2_O: 20.0; Ca–lactate: 356.5; CuCl: 0.2; AlCl_3_·6H_2_O: 0.15; KI: 0.15; Na_2_Se_2_O_3_: 0.01; MnSO_4_·H_2_O: 2.0; CoCl_2_·6H_2_O: 1.0.

**Table 2:** Amino acid profiles (% of the diet) of the experimental diets.

	Ingredients		Requirement	Experimental diets			
	FM	CGM		Con	CG20	CG40	CG60
Essential amino acids (EAA, %)							
Arginine	4.08	2.15	2.37^c^	2.58	2.39	2.17	2.01
Histidine	1.69	1.32	—	1.38	1.25	1.12	1.00
Isoleucine	2.69	2.44	—	1.70	1.69	1.67	1.66
Leucine	5.01	10.95	—	3.82	4.12	4.41	4.74
Lysine	5.56	1.22	1.79^d^	3.68	3.23	2.78	2.33
Phenylalanine	2.78	4.02	—	2.12	2.32	2.51	2.74
Threonine	3.10	2.28	—	2.30	2.26	2.21	2.16
Tryptophan	1.14	0.28	—	0.36	0.32	0.28	0.25
Valine	3.37	2.92	0.90^e^	2.30	2.29	2.27	2.26
*∑*EAA^a^	30.55	29.33	—	20.24	19.87	19.42	19.15
Nonessential amino acids (NEAA, %)							
Alanine	4.16	4.67	—	2.76	3.05	3.32	3.64
Aspartic acid	6.07	3.22	—	4.95	4.77	4.60	4.42
Glutamic acid	8.34	11.36	—	6.34	6.52	6.70	6.89
Glycine	3.76	1.43	—	2.94	2.68	2.43	2.18
Proline	2.74	5.14	—	2.16	2.66	3.15	3.72
Serine	2.58	2.72	—	1.72	1.87	2.01	2.14
Tyrosine	1.82	2.31	—	1.39	1.57	1.75	1.93
*∑*NEAA^b^	29.47	30.85	—	22.26	23.12	23.96	24.92

^a^ *∑*EAA: Total essential amino acids.

^b^ *∑*NEAA: Total nonessential amino acids.

^c^Arginine.

^d^lysine.

^e^valine requirements of red sea bream were obtained from studies by Duncan [32], Rahimnejad and Lee [33], and Forster and Ogata [34], respectively.

**Table 3:** Fatty acid profiles (% of total fatty acids) of the experimental diets.

	Ingredients		Experimental diets			
	FM	CGM	Con	CG20	CG40	CG60
C14:0	5.01	0.07	2.49	2.28	2.07	1.85
C16:0	23.06	12.83	15.72	15.17	14.62	14.17
C18:0	8.05	2.12	4.77	4.45	4.32	4.05
C20:0	0.98	0.49	0.76	0.74	0.72	0.71
C22:0	0.30	0.33	0.34	0.35	0.37	0.38
C24:0	0.68	0.00	0.48	0.40	0.36	0.31
*∑*SFA^a^	38.08	15.84	24.56	23.39	22.46	21.47
C14:1*n*−7	0.23	0.00	0.10	0.08	0.07	0.06
C15:1*n*−5	0.15	0.02	0.10	0.09	0.08	0.06
C16:1*n*−7	5.47	0.23	3.14	2.92	2.70	2.47
C17:1*n*−7	0.78	0.05	0.38	0.34	0.30	0.26
C18:1*n*−9	23.30	25.88	30.22	30.53	30.83	31.16
C20:1*n*−9	1.01	0.01	1.01	0.99	0.97	0.95
C22:1*n*−9	0.19	0.00	0.38	0.36	0.35	0.33
C24:1*n*−9	2.69	0.00	1.08	0.99	0.91	0.82
*∑*MUFA^b^	33.82	26.19	36.41	36.30	36.21	36.11
C18:2*n*−6	1.89	53.66	24.10	26.51	28.64	30.63
C18:3*n*−3	0.70	2.51	3.74	3.81	3.88	3.95
C18:3*n*−6	0.30	0.01	0.39	0.38	0.37	0.35
C20:2*n*−6	0.07	0.05	0.07	0.06	0.06	0.05
C20:3*n*−3	0.17	0.03	0.02	0.02	0.02	0.01
C20:3*n*−6	0.08	0.00	0.02	0.02	0.01	0.01
C20:4*n*−6	2.44	0.00	0.73	0.64	0.55	0.46
C20:5*n*−3	7.06	0.00	2.82	2.56	2.25	1.89
C22:2*n*−6	0.60	0.52	0.39	0.38	0.37	0.35
C22:6*n*−3	14.71	0.00	4.87	4.27	3.65	3.05
DHA:EPA ratio	2.08	0.00	1.73	1.67	1.62	1.61
*∑n*−3 HUFA^c^	21.94	0.03	7.71	6.85	5.92	4.95
Unknown	0.08	1.19	1.97	1.66	1.53	1.67

^a^ *∑*SFA: Total saturated fatty acids.

^b^ *∑*MUFA: Total monounsaturated fatty acids.

^c^ *∑n*−3 HUFA: Total *n*−3 highly unsaturated fatty acids.

**Table 4:** Survival (%), feed conversion ratio (FCR), protein efficiency ratio (PER), protein retention (PR, %), condition factor (*K*), viscerosomatic index (VSI, %), and hepatosomatic index (HSI, %) of red sea bream fed the experimental diets substituting various levels of fish meal with corn gluten meal.

Dependent variables	Experimental diets				*p*-Value	Orthogonal polynomial contrast			Regression analysis		
	Con	CG20	CG40	CG60		Linear	Quadratic	Cubic	Equation	*R* ^2^	*p*-Value
IBW (g/fish)	8.59 ± 0.013	8.63 ± 0.035	8.61 ± 0.013	8.57 ± 0.013	*p* > 0.3	—	—	—	—	—	—
FBW (g/fish)	41.53 ± 0.667^a^	39.82 ± 1.764^a^	31.24 ± 1.528^b^	27.66 ± 1.528^c^	*p* < 0.0001	0.0001	0.3183	0.0170	*Y* = – 5.021097*X* + 47.6162	0.8991	*p* < 0.0001
Survival (%)	91.33 ± 1.333	91.33 ± 3.528	90.00 ± 3.055	86.00 ± 3.055	*p* > 0.5	0.2704	0.4563	0.9657	—	—	—
FCR^1^	1.03 ± 0.004^b^	1.08 ± 0.015^b^	1.29 ± 0.044^a^	1.39 ± 0.044^a^	*p* < 0.0002	0.0001	0.4897	0.0819	*Y* = 0.127333*X* + 0.8767	0.8621	*p* < 0.0001
PER^2^	1.77 ± 0.022^a^	1.73 ± 0.065^a^	1.45 ± 0.084^b^	1.37 ± 0.048^b^	*p* < 0.003	0.0005	0.7250	0.1392	*Y* = – 0.149000*X* + 1.9517	0.7435	*p* < 0.0004
PR^3^ (%)	28.32 ± 0.681^a^	27.87 ± 0.697^a^	23.27 ± 1.318^b^	22.20 ± 0.992^b^	*p* < 0.004	0.0006	0.7434	0.1128	*Y* = – 0.023857*X* + 0.3166	0.7208	*p* < 0.0006
*K* ^4^	1.86 ± 0.027	1.91 ± 0.017	1.87 ± 0.008	1.85 ± 0.036	*p* > 0.2	0.5188	0.1194	0.3494	—	—	—
VSI^5^ (%)	8.76 ± 0.297	9.02 ± 0.360	9.58 ± 0.117	9.35 ± 0.199	*p* > 0.2	0.0810	0.3668	0.3742	—	—	—
HSI^6^ (%)	2.73 ± 0.066^b^	2.72 ± 0.025^b^	3.00 ± 0.031^a^	3.05 ± 0.080^a^	*p* < 0.005	0.0011	0.6100	0.0638	*Y* = 0.001237*X* + 0.0257	0.6583	*p* < 0.002

*Note:* Values (means of triplicate ± SE) in the same row sharing the same superscript letter are not significantly different (*p* > 0.05).

Abbreviations: FBW, final body weight; IBW, initial body weight.

^1^Feed conversion ratio (FCR) = total feed consumption (g)/(total final weight (g) − total initial weight (g) + total weight of dead fish (g)).

^2^Protein efficiency ratio (PER) = weight gain of fish (g/fish)/protein consumption of fish (g/fish).

^3^Protein retention (PR, %) = protein gain of fish (g/fish) × 100/protein consumption of fish (g/fish).

^4^Condition factor (*K*) = body weight of fish (g) × 100/total length of fish (cm)^3^.

^5^Viscerosomatic index (VSI, %) = viscera weight of fish (g) × 100/body weight of fish (g).

^6^Hepatosomatic index (HSI, %) = liver weight of fish (g) × 100/body weight of fish (g).

**Table 5:** Blood chemistry of red sea bream fed experimental diets.

Dependent variables	Experimental diets				*p*-Value	Orthogonal polynomial contrast		
	Con	CG20	CG40	CG60		Linear	Quadratic	Cubic
Plasma parameters								
AST (U/L)	50.89 ± 1.124	48.89 ± 1.730	48.44 ± 0.463	53.33 ± 1.836	*p* > 0.2	0.5430	0.1932	0.7365
ALT (U/L)	9.11 ± 0.723	9.11 ± 0.714	8.67 ± 0.588	8.67 ± 0.509	*p* > 0.8	0.7291	1.0000	0.8621
ALP (U/L)	168.89 ± 1.847	171.44 ± 3.625	171.56 ± 3.304	174.67 ± 2.697	*p* > 0.6	0.4667	0.9579	0.8175
T-BIL (U/L)	0.89 ± 0.056	0.80 ± 0.073	0.92 ± 0.034	0.89 ± 0.045	*P* > 0.8	0.7770	0.7735	0.4051
T-CHO (mg/dL)	251.56 ± 3.681	252.78 ± 4.002	256.00 ± 2.728	248.56 ± 4.590	*P* > 0.6	0.8500	1.0000	0.5997
TG (mg/dL)	408.67 ± 12.894	416.11 ± 2.158	405.11 ± 12.977	419.11 ± 16.123	*P* > 0.1	0.8356	0.8810	0.6590
TP (g/dL)	4.68 ± 0.101	5.02 ± 5.120	4.88 ± 0.089	4.79 ± 0.050	*p* > 0.4	0.8009	0.2178	0.4738
ALB (g/dL)	1.06 ± 0.094	1.01 ± 0.061	0.97 ± 0.048	1.08 ± 0.114	*p* > 0.2	0.9735	0.6062	0.8164
Serum parameters								
SOD (ng/mL)	3.91 ± 0.289	4.27 ± 0.457	4.27 ± 0.584	4.53 ± 0.277	*p* > 0.9	0.5061	0.9326	0.8223
Lysozyme activity (U/mL)	34.93 ± 1.702	48.51 ± 4.774	47.08 ± 4.056	42.35 ± 4.823	*p* > 0.5	0.5303	0.2330	0.7213

*Note:* Values (means of triplicate ± SE).

Abbreviations: ALB, albumin; ALP, alkaline phosphatase; ALT, alanine aminotransferase; AST, aspartate aminotransferase; T-BIL, total bilirubin; T-CHO, total cholesterol; TG, triglyceride; TP, total protein.

**Table 6:** Chemical composition (%, wet weight) of the whole-body red sea bream fed the experimental diets.

Dependent variables	Experimental diets				*p*-Value	Orthogonal polynomial contrast		
	Con	CG20	CG40	CG60		Linear	Quadratic	Cubic
Moisture	69.40 ± 0.976	69.18 ± 0.666	69.27 ± 0.579	69.21 ± 0.113	*p* > 0.9	0.8834	0.9065	0.8713
Crude protein	16.07 ± 0.376	16.17 ± 0.183	16.13 ± 0.083	16.24 ± 0.109	*p* > 0.9	0.6592	0.9707	0.7804
Crude lipid	8.70 ± 0.268	8.98 ± 0.208	8.93 ± 0.412	8.85 ± 0.069	*p* > 0.8	0.7240	0.5148	0.8218
Ash	4.61 ± 0.239	4.32 ± 0.095	4.47 ± 0.223	4.63 ± 0.127	*p* > 0.6	0.7906	0.2448	0.6060

*Note:* Values (means of triplicate ± SE).

**Table 7:** Amino acid profiles (%, wet weight) of the whole-body red sea bream fed the experimental diets.

	Experimental diets				*p*-Value	Orthogonal polynomial contrast			Regression analysis		
	Con	CG20	CG40	CG60		Linear	Quadratic	Cubic	Equation	*R* ^2^	*p*-Value
Essential amino acids (%)											
Arginine	1.00 ± 0.032^a^	0.93 ± 0.026^a^	0.80 ± 0.029^b^	0.72 ± 0.029^b^	*p* < 0.0006	0.0001	0.7809	0.3689	*Y* = – 0.096333*X* + 1.1033	0.8602	*p* < 0.005
Histidine	0.34 ± 0.026	0.33 ± 0.020	0.32 ± 0.017	0.32 ± 0.017	*p* > 0.8	0.4283	0.6957	0.9720	—	—	—
Isoleucine	0.52 ± 0.017	0.51 ± 0.017	0.50 ± 0.015	0.48 ± 0.017	*p* > 0.4	0.1227	0.6996	0.7952	—	—	—
Leucine	1.12 ± 0.020	1.10 ± 0.017	1.11 ± 0.020	1.12 ± 0.020	*p* > 0.7	0.9412	0.3369	0.8251	—	—	—
Lysine	1.26 ± 0.020^a^	1.20 ± 0.017^a^	1.09 ± 0.032^b^	1.05 ± 0.029^b^	*p* < 0.002	0.0002	0.6122	0.2950	*Y* = – 0.075333*X* + 1.3383	0.8233	*p* < 0.0001
Phenylalanine	0.59 ± 0.026	0.61 ± 0.026	0.63 ± 0.026	0.65 ± 0.023	*p* > 0.3	0.1009	0.9492	0.9772	—	—	—
Threonine	0.72 ± 0.020	0.71 ± 0.017	0.69 ± 0.020	0.68 ± 0.020	*p* > 0.4	0.1403	0.8049	0.7407	—	—	—
Tryptophan	0.10 ± 0.017	0.09 ± 0.015	0.08 ± 0.015	0.07 ± 0.015	*p* > 0.6	0.2242	0.9158	0.9623	—	—	—
Valine	0.67 ± 0.020	0.66 ± 0.020	0.65 ± 0.020	0.64 ± 0.020	*p* > 0.7	0.3022	0.7508	1.0000	—	—	—
Nonessential amino acids (%)											
Alanine	1.13 ± 0.023	1.07 ± 0.020	1.10 ± 0.017	1.13 ± 0.017	*p* > 0.1	0.7143	0.0449	0.2881	—	—	—
Aspartic acid	1.49 ± 0.020	1.45 ± 0.020	1.44 ± 0.019	1.43 ± 0.020	*p* > 0.2	0.0619	0.3923	0.9149	—	—	—
Glutamic acid	2.11 ± 0.017	2.07 ± 0.017	2.09 ± 0.017	2.11 ± 0.017	*p* > 0.3	0.8028	0.1215	0.4609	—	—	—
Glycine	1.18 ± 0.020	1.16 ± 0.021	1.14 ± 0.020	1.13 ± 0.020	*p* > 0.3	0.1093	0.6919	0.9149	—	—	—
Proline	0.72 ± 0.015	0.73 ± 0.017	0.75 ± 0.017	0.76 ± 0.017	*p* > 0.3	0.0790	0.9228	0.8287	—	—	—
Serine	0.72 ± 0.017	0.74 ± 0.020	0.76 ± 0.020	0.79 ± 0.015	*p* > 0.1	0.0212	0.7245	0.6938	—	—	—
Tyrosine	0.44 ± 0.020	0.43 ± 0.020	0.42 ± 0.017	0.41 ± 0.021	*p* > 0.7	0.3208	0.8700	0.8836	—	—	—

*Note:* Values (means of triplicate ± SE) in the same row sharing the same superscript letter are not significantly different (*p* > 0.05).

**Table 8:** Fatty acid profiles (%, total fatty acids) of the whole-body red sea bream fed the experimental diets.

	Experimental diets				*p*-Value	Orthogonal polynomial contrast		
	Con	CG20	CG40	CG60		Linear	Quadratic	Cubic
C14:0	2.66 ± 0.029	2.62 ± 0.032	2.56 ± 0.061	2.50 ± 0.115	*p* > 0.4	0.1075	0.9064	0.9079
C16:0	15.59 ± 0.107	15.48 ± 0.118	15.34 ± 0.289	15.27 ± 0.300	*p* > 0.7	0.2966	0.9307	0.9175
C18:0	4.58 ± 0.049	4.53 ± 0.046	4.47 ± 0.029	4.42 ± 0.032	*p* > 0.07	0.0139	1.0000	0.9424
C20:0	1.07 ± 0.064	1.05 ± 0.064	1.02 ± 0.064	1.00 ± 0.058	*p* > 0.8	0.3946	0.9793	0.9536
C22:0	0.80 ± 0.018	0.82 ± 0.020	0.84 ± 0.020	0.86 ± 0.020	*p* > 0.2	0.0664	1.0000	0.7693
*∑*SFA^a^	24.71 ± 0.139	24.50 ± 0.090	24.23 ± 0.115	24.04 ± 0.370	*p* > 0.2	0.0642	0.9676	0.8942
C14:1*n*−7	0.10 ± 0.009	0.10 ± 0.006	0.09 ± 0.006	0.08 ± 0.003	*p* > 0.06	0.0121	0.4458	0.9078
C15:1*n*−5	0.07 ± 0.009	0.06 ± 0.009	0.03 ± 0.012	0.04 ± 0.009	*p* > 0.08	0.0083	0.7802	0.9005
C16:1*n*−7	4.52 ± 0.058	4.48 ± 0.058	4.46 ± 0.061	4.41 ± 0.061	*p* > 0.6	0.2271	0.9132	0.8266
C17:1*n*−7	0.63 ± 0.043	0.61 ± 0.046	0.58 ± 0.043	0.55 ± 0.043	*p* > 0.6	0.2346	0.9416	0.8957
C18:1*n*−9	31.79 ± 0.400	31.98 ± 0.258	32.05 ± 0.228	32.09 ± 0.462	*p* > 0.9	0.5521	0.8324	0.9442
C20:1*n*−9	3.44 ± 0.229	3.22 ± 0.078	3.19 ± 0.087	3.15 ± 0.087	*p* > 0.5	0.1715	0.5031	0.7458
C22:1*n*−9	0.05 ± 0.020	0.04 ± 0.020	0.04 ± 0.017	0.03 ± 0.015	*p* > 0.8	0.4603	0.9295	0.9055
C24:1*n*−9	1.19 ± 0.020	1.17 ± 0.038	1.11 ± 0.035	1.05 ± 0.035	*p* > 0.05	0.0094	0.5553	0.7563
*∑*MUFA^b^	41.79 ± 0.566	41.66 ± 0.269	41.54 ± 0.280	41.40 ± 0.430	*p* > 0.9	0.4935	0.9873	0.9858
C18:2*n*−6	16.37 ± 0.214	16.65 ± 0.274	17.12 ± 0.638	17.54 ± 0.595	*p* > 0.3	0.0951	0.8852	0.9191
C18:3*n*−3	1.50 ± 0.087	1.57 ± 0.066	1.65 ± 0.058	1.71 ± 0.069	*p* > 0.2	0.0543	0.9636	0.9026
C18:3*n*−6	0.17 ± 0.023	0.17 ± 0.017	0.16 ± 0.015	0.15 ± 0.017	*p* > 0.8	0.4397	0.7256	1.0000
C20:2*n*−6	0.13 ± 0.015	0.12 ± 0.020	0.11 ± 0.023	0.10 ± 0.023	*p* > 0.7	0.3395	0.8752	0.8882
C20:3*n*−3	0.25 ± 0.032	0.28 ± 0.029	0.27 ± 0.029	0.25 ± 0.032	*p* > 0.8	0.9431	0.4342	0.7940
C20:3*n*−6	0.42 ± 0.038	0.41 ± 0.035	0.39 ± 0.038	0.39 ± 0.032	*p* > 0.5	0.5725	0.8556	0.7761
C20:4*n*−6	0.94 ± 0.028	0.89 ± 0.049	0.93 ± 0.052	0.89 ± 0.055	*p* > 0.7	0.5472	0.9457	0.4547
C20:5*n*−3	4.72 ± 0.175	4.67 ± 0.115	4.63 ± 0.124	4.59 ± 0.130	*p* > 0.9	0.5094	1.0000	0.9750
C22:2*n*−6	0.45 ± 0.058	0.44 ± 0.061	0.42 ± 0.058	0.40 ± 0.061	*p* > 0.9	0.5545	0.9348	0.9319
C22:6*n*−3	6.35 ± 0.099	6.33 ± 0.123	6.30 ± 0.146	6.28 ± 0.196	*p* > 0.9	0.5545	0.9348	0.9319
*∑n*−3 HUFA^c^	11.07 ± 0.210	11.00 ± 0.032	10.93 ± 0.032	10.86 ± 0.066	*p* > 0.6	0.6929	1.0000	0.9604
Unknown	2.20 ± 0.317	2.29 ± 0.093	2.25 ± 0.057	2.26 ± 0.306	—	—	—	—

*Note:* Values (means of triplicate ± SE).

^a^ *∑*SFA: Total saturated fatty acids.

^b^ *∑*MUFA: Total monounsaturated fatty acids.

^c^ *∑n*−3 HUFA: Total *n*−3 highly unsaturated fatty acids.

**Table 9:** Substitutability of corn gluten meal for fish meal (FM) in diets of various marine fish.

Fish species (scientific name)	Recommended FM substitution levels of diet (%)	FM inclusion levels in the FM–basal diet (%)	Fish size	Criteria measured	Supplemented essential amino acids	References
Puffer (*Takifugu fasciatus*)	15	54	Grown from 41 to 81 g	FBW and WG	—	[40]

Asian seabass (*Lates calcarifer*)	29	35	Grown from 22 to 39 g	FBW and PR	—	[9]

Red sea bream (*Pagrus major*)	20	55	Grown from 9 to 42 g	WG, SGR, FCR, PER, and PR	—	This study
	30	50	Grown from 53 to 89 g	FBW, WG, and daily growth rate	—	[22]
	70	50	Grown from 280 to 751 g	FBW	—	

Gilthead sea bream (*Sparus aurata*)	60	62	Grown from 8 to 31 g	FBW	—	[39]

Olive flounder (*Paralichthys olivaceus*)	40	75	Grown from 8 to 33 g	FBW, WG, FCR, and PER	Arginine, lysine, and tryptophan	[14]

Seabass (*Lateolabrax japonicus*)	60	52	Grown from 18 to 80 g	SGR	Arginine, isoleucine, lysine, methionine, valine, and threonine	[41]

Spotted rose snapper (*Lutjanus guttatus*)	60	55	Grown from 31 to 119 g	WG, SGR, FCR, and PER	Arginine and lysine	[1]

Abbreviations: FBW, final body weight; FCR, feed conversion ratio; PER, protein efficiency ratio; PR, protein retention; SGR, specific growth rate; WG, weight gain.

## Data Availability

The data that support the findings of this study are available from the corresponding author upon reasonable request.

## References

[1] Hernández C., Lizárraga-Velázquez C. E., Contreras-Rojas D. (2021). Fish Meal Replacement by Corn Gluten in Feeds for Juvenile Spotted Rose Snapper (*Lutjanus guttatus*): Effect on Growth Performance, Feed Efficiency, Hematological Parameters, Protease Activity, Body Composition, and Nutrient Digestibility. *Aquaculture*.

[2] Perez-Velazquez M., Gatlin D. M., González-Félix M. L. (2019). Effect of Fishmeal and Fish Oil Replacement by Algal Meals on Biological Performance and Fatty Acid Profile of Hybrid Striped Bass (*Morone crhysops*♀× *M. saxatilis*♂). *Aquaculture*.

[3] Oliva-Teles A., Enes P., Peres H. (2015). Replacing Fishmeal and Fish Oil in Industrial Aquafeeds for Carnivorous Fish. *Feed and Feeding Practices in Aquaculture*.

[4] Index mundi (2024). Fish-Meal Production by Country in 1000 MT.

[5] Asche F., Oglend A., Tveteras S. (2013). Regime Shifts in the Fish Meal/Soybean Meal Price Ratio. *Journal of Agricultural Economics*.

[6] Jannathulla R., Rajaram V., Kalanjiam R., Ambasankar K., Muralidhar M., Dayal J. S. (2019). Fishmeal Availability in the Scenarios of Climate Change: Inevitability of Fishmeal Replacement in Aquafeeds and Approaches for the Utilization of Plant Protein Sources. *Aquaculture Research*.

[7] Hardy R. W. (2010). Utilization of Plant Proteins in Fish Diets: Effects of Global Demand and Supplies of Fishmeal. *Aquaculture Research*.

[8] Index mundi (2024). Fish-Meal Monthly Price.

[9] Hussain S. M., Bano A. A., Ali S. (2024). Substitution of Fishmeal: Highlights of Potential Plant Protein Sources for Aquaculture Sustainability. *Heliyon*.

[10] Nandakumar S., Ambasankar K., Ali S. S. R., Syamadayal J., Vasagam K. (2017). Replacement of Fish Meal With Corn Gluten Meal in Feeds for Asian Seabass (*Lates calcarifer*). *Aquaculture International*.

[11] Biswas A., Araki H., Sakata T., Nakamori T., Takii K. (2019). Optimum Fish Meal Replacement by Soy Protein Concentrate From Soymilk and Phytase Supplementation in Diet of Red Sea Bream, *Pagrus major*. *Aquaculture*.

[12] Dossou S., Koshio S., Ishikawa M. (2019). Effects of Replacing Fishmeal With Fermented and Non-Fermented Rapeseed Meal on the Growth, Immune and Antioxidant Responses of Red Sea Bream (*Pagrus major*). *Aquaculture Nutrition*.

[13] Ng W.-K., Leow T.-C., Yossa R. (2019). Effect of Substituting Fishmeal With Corn Protein Concentrate on Growth Performance, Nutrient Utilization and Skin Coloration in Red Hybrid Tilapia, *Oreochromis* sp. *Aquaculture Nutrition*.

[14] Zhong G., Hua X., Yuan K., Zhou H. (2011). Effect of CGM on Growth Performance and Digestibility in Puffer (*Takifugu fasciatus*). *Aquaculture International*.

[15] Kikuchi K. (1999). Partial Replacement of Fish Meal With Corn Gluten Meal in Diets for Japanese Flounder *Paralichthys olivaceus*. *Journal of the World Aquaculture Society*.

[16] Lee S., Yoo J., Lee J. Y. (1996). The use of Soybean Meal, Corn Gluten Meal, Meat Meal, Meat and Bone Meal, or Blood Meal as a Dietary Protein Source Replacing Fish Meal in Korean Rockfish (*Sebastes schlegeli*). *Korean Journal of Animal Nutrition and Feedstuffs*.

[17] Herault M., Gunathilaka B. E., Fournier V., Bris H. Le, Lee K., Sadoul B. (2023). Aquatic Product Hydrolysates Increase Rearing Performance in Red Seabream (*Pagrus major*), Fed a Low Fish Meal Diet, in Both Controlled and Stressed Conditions: From Growth to Stress Responses. *Aquaculture*.

[18] KOSIS (2024). Korean Statistical Information Service.

[19] Seong T., Kitagima R., Haga Y., Satoh S. (2020). Non-Fish Meal, Non-Fish Oil Diet Development for Red Sea Bream, *Pagrus major*, With Plant Protein and Graded Levels of *Schizochytrium* sp.: Effect on Growth and Fatty Acid Composition. *Aquaculture Nutrition*.

[20] Seong T., Matsutani H., Haga Y., Kitagima R., Satoh S. (2019). First Step of Non-Fish Meal, Non-Fish Oil Diet Development for Red Seabream, (*Pagrus major*), With Plant Protein Sources and Microalgae *Schizochytrium* sp. *Aquaculture Research*.

[21] Seong T., Uno Y., Kitagima R., Kabeya N., Haga Y., Satoh S. (2021). Microalgae as Main Ingredient for Fish Feed: Non-Fish Meal and Non-Fish Oil Diet Development for Red Sea Bream, *Pagrus major*, by Blending of Microalgae *Nannochloropsis*, *Chlorella* and *Schizochytrium*. *Aquaculture Research*.

[22] Gunathilaka B. E., Jeong S., Cho M. (2023). Effects of Dietary Fish Meal Replacement With Alternative Protein Ingredients and Their Combinations on Growth Performance, Feed Utilization, Fillet Composition, and Biochemical Parameters of Red Seabream (*Pagrus major*). *Aquaculture Nutrition*.

[23] Takagi S., Hosokawa H., Shimeno S., Ukawa M. (2000). Utilization of Corn Gluten Meal in a Diet for Red Sea Bream *Pagrus major*. *Nippon Suisan Gakkaishi*.

[24] Sim Y. J., Cho S. H. (2025). Effect of Partial or Complete Substitution of Fish Meal by Meat Meal in the Feed of Red Sea Bream (*Pagrus major*) on the Growth Performance and Feed Utilization. *Aquaculture Nutrition*.

[25] Kim K., Jang J. W., Kim K., Lee B., Hur S. W., Han H. (2018). Tuna By-Product Meal as a Dietary Protein Source Replacing Fishmeal in Juvenile Korean Rockfish *Sebastes schlegeli*. *Fisheries and Aquatic Sciences*.

[26] Li R., Cho S. H. (2023). Substitution Impact of Tuna by-Product Meal for Fish Meal in the Diets of Rockfish (*Sebastes schlegeli*) on Growth and Feed Availability. *Animals*.

[27] Takeuchi T., Shiina Y., Watanabe T. (1991). Suitable Protein and Lipid Levels in Diet for Fingerlings of Red Sea Bream *Pagrus major*. *Nippon Suisan Gakkaishi*.

[28] Baek S. I., Cho S. H. (2024). Dietary Replacement Effect of Fish Meal by Tuna by-Product Meal on Growth and Feed Availability of Red Sea Bream (*Pagrus major*). *Animals*.

[29] Lee M. J., Kim J., Baek S. I., Cho S. H. (2023). Substitution Effect of Fish Meal With Meat Meal in Diet on Growth Performance, Feed Consumption, Feed Utilization, Chemical Composition, Hematology, and Innate Immune Responses of Rockfish (*Sebastes schlegeli*). *Aquaculture*.

[30] AOAC (1990). *Official Methods of Analysis*.

[31] Folch J., Lees M., Stanley G. H. S. (1957). A Simple Method for the Isolation and Purification of Total Lipids From Animal Tissues. *Journal of Biological Chemistry*.

[32] Duncan D. B. (1955). Multiple Range and Multiple F Tests. *Biometrics*.

[33] Rahimnejad S., Lee K.-J. (2014). Dietary Arginine Requirement of Juvenile Red Sea Bream *Pagrus major*. *Aquaculture*.

[34] Forster I., Ogata H. Y. (1998). Lysine Requirement of Juvenile Japanese Flounder *Paralichthys olivaceus* and Juvenile Red Sea Bream *Pagrus major*. *Aquaculture*.

[35] Rahimnejad S., Lee K. (2013). Dietary Valine Requirement of Juvenile Red Sea Bream *Pagrus major*. *Aquaculture*.

[36] Gunathilaka B. E., Khosravi S., Shin J. (2021). Evaluation of Shrimp Protein Hydrolysate and Krill Meal Supplementation in Low Fish Meal Diet for Red Seabream (*Pagrus major*). *Fisheries and Aquatic Sciences*.

[37] Lee S.-M., Mohammadi Azarm H., Chang K. H. (2016). Effects of Dietary Inclusion of Fermented Soybean Meal on Growth, Body Composition, Antioxidant Enzyme Activity and Disease Resistance of Rockfish (*Sebastes schlegeli*). *Aquaculture*.

[38] Sevgili H., Kurtoğlu A., Oikawa M. (2015). A Combination of Corn Gluten and Soybean Meal as a Substitute for Fishmeal in Diets of Turbot (*Scophthalmus maximus* Linnaeus, 1758) in Brackish Water. *Journal of Applied Ichthyology*.

[39] Wang L., Yin N., Sagada G. (2020). Partial Replacement of Fishmeal With Corn Gluten Meal, Pea Protein Isolate and Their Mixture in Diet of Black Sea Bream (*Acanthopagrus schlegelii*) Juveniles: Effects on Growth Performance, Feed Utilization and Haematological Parameters. *Aquaculture Research*.

[40] Lin H., Deng Y., Zhu D. (2023). Effects of Partially Replacing Fishmeal With Corn Gluten Meal on Growth, Feed Utilization, Digestive Enzyme Activity, and Apparent Nutrient Digestibility for Juvenile White Shrimp, *Litopenaeus vannamei*. *Frontiers in Veterinary Science*.

[41] Pereira T. G., Oliva-Teles A. (2003). Evaluation of Corn Gluten Meal as a Protein Source in Diets for Gilthead Sea Bream (*Sparus aurata* L.) Juveniles. *Aquaculture Research*.

[42] Vagner M., Zambonino-Infante J., Mazurais D. (2014). Reduced n-3 Highly Unsaturated Fatty Acids Dietary Content Expected With Global Change Reduces the Metabolic Capacity of the Golden Grey Mullet. *Marine Biology*.

[43] Houston S. J. S., Karalazos V., Tinsley J., Tocher D. R., Glencross B. D., Monroig O. (2022). A Comparison of Regression Models for Defining EPA+ DHA Requirements Using the Gilthead Seabream (*Sparus aurata*) as a Model Species. *Aquaculture*.

[44] Sargent J., Bell G., McEvoy L., Tocher D., Estevez A. (1999). Recent Developments in the Essential Fatty Acid Nutrition of Fish. *Aquaculture*.

[45] Men K., Ai Q., Mai K., Xu W., Zhang Y., Zhou H. (2014). Effects of Dietary Corn Gluten Meal on Growth, Digestion and Protein Metabolism in Relation to IGF-I Gene Expression of Japanese Seabass, *Lateolabrax japonicus*. *Aquaculture*.

[46] Baek S. I., Jeong H. S., Cho S. H. (2023). Replacement Effect of Fish Meal by Plant Protein Sources in Olive Flounder (*Paralichthys olivaceus*) Feeds With an Addition of Jack Mackerel Meal on Growth, Feed Availability, and Biochemical Composition. *Aquaculture Nutrition*.

[47] Kim J., Cho S. H. (2024). Substitution Effect of Fish Meal With Various Plant Protein Sources on Growth Performance and Feed Utilization in Rockfish (*Sebastes schlegeli*) Diets including Jack Mackerel Meal Used as Feed Stimulants. *Frontiers in Marine Science*.

[48] Carr W. E. S., Netherton J. C., Gleeson R. A., Derby C. D. (1996). Stimulants of Feeding Behavior in Fish: Analyses of Tissues of Diverse Marine Organisms. *The Biological Bulletin*.

[49] Regost C., Arzel J., Kaushik S. J. (1999). Partial or Total Replacement of Fish Meal by Corn Gluten Meal in Diet for Turbot (*Psetta maxima*). *Aquaculture*.

[50] Bu X., Lian X., Zhang Y. (2018). Effects of Replacing Fish Meal With Corn Gluten Meal on Growth, Feed Utilization, Nitrogen and Phosphorus Excretion and IGF-I Gene Expression of Juvenile *Pseudobagrus ussuriensis*. *Aquaculture Research*.

[51] Nash R. D. M., Valencia A. H., Geffen A. J. (2006). The Origin of Fulton’s Condition Factor-Setting the Record Straight. *Fisheries*.

[52] Chellappa S., Huntingford F. A., Strang R. H. C., Thomson R. Y. (1995). Condition Factor and Hepatosomatic Index as Estimates of Energy Status in Male Three-Spined Stickleback. *Journal of Fish Biology*.

[53] Alam M. S., Liang X.-F., Liu L. (2020). Indirect Effect of Different Dietary Protein to Energy Ratio of Bait Fish Mori Diets on Growth Performance, Body Composition, Nitrogen Metabolism and Relative AMPK & mTOR Pathway Gene Expression of Chinese Perch. *Aquaculture Reports*.

[54] Kamalam B. S., Panserat S. (2016). Carbohydrates in Fish Nutrition. *International Aquafeed*.

[55] Ye H., Xu M., Chen L. (2019). Effects of Dietary Plant Protein Sources Influencing Hepatic Lipid Metabolism and Hepatocyte Apoptosis in Hybrid Grouper (*Epinephelus lanceolatus*♂×*Epinephelus fuscoguttatus*♀). *Aquaculture*.

[56] Ye J., Liu X., Wang Z., Wang K. (2011). Effect of Partial Fish Meal Replacement by Soybean Meal on the Growth Performance and Biochemical Indices of Juvenile Japanese Flounder *Paralichthys olivaceus*. *Aquaculture International*.

[57] Kohjima M., Enjoji M., Higuchi N. (2007). Re-Evaluation of Fatty Acid Metabolism-Related Gene Expression in Nonalcoholic Fatty Liver Disease. *International Journal of Molecular Medicine*.

[58] Xiang X., Ji R., Han S. (2024). Differences in Diacylglycerol Acyltransferases Expression Patterns and Regulation Cause Distinct Hepatic Triglyceride Deposition in Fish. *Communications Biology*.

[59] Luan X., Li X., He J. (2025). Effects of Arginine Family Amino Acids Supplementation on Growth, Whole-Body Amino Acid Profiles, Antioxidant Capacity, and Gene Expression of Juvenile Largemouth Bass (*Micropterus salmoides*). *Aquaculture*.

[60] Huang X., Chen F., Sang Y., Li Y., Xie D. (2024). Medium-Short Chain Fatty Acids Cannot Spare the Essential Fatty Acids of Low Marine Diets in Golden Pompano (*Trachinotus ovatus*). *Aquaculture Reports*.

[61] Pham H. D., Siddik M. A. B., Fotedar R. (2020). Substituting Fishmeal With Lupin *Lupinus angustifolius* Kernel Meal in the Diets of Cobia *Rachycentron canadum*: Effects on Growth Performance, Nutrient Utilization, Haemato-Physiological Response, and Intestinal Health. *Animal Feed Science and Technology*.

[62] Zaitsev S. (2016). Dynamic Surface Tension Measurements as General Approach to the Analysis of Animal Blood Plasma and Serum. *Advances in Colloid and Interface Science*.

[63] Jomova K., Alomar S. Y., Alwasel S. H., Nepovimova E., Kuca K., Valko M. (2024). Several Lines of Antioxidant Defense Against Oxidative Stress: Antioxidant Enzymes, Nanomaterials With Multiple Enzyme-Mimicking Activities, and Low-Molecular-Weight Antioxidants. *Archives of Toxicology*.

[64] Ragland S. A., Criss A. K. (2017). From Bacterial Killing to Immune Modulation: Recent Insights into the Functions of Lysozyme. *PLoS Pathogens*.

[65] Li W., Xu B., Wei F., Li S., Wang S., Wen X. (2020). Effects of Partial Substitution of Dietary Fishmeal by Fermented Soybean Meal on Growth, Amino Acid and Protein Metabolism of Juvenile *Nibea diacanthus*. *Aquaculture Nutrition*.

[66] Liu H., Zhu X., Yang Y., Han D., Jin J., Xie S. (2016). Effect of Substitution of Dietary Fishmeal by Soya Bean Meal on Different Sizes of Gibel Carp (*Carassius auratus gibelio*): Nutrient Digestibility, Growth Performance, Body Composition and Morphometry. *Aquaculture Nutrition*.

[67] Monge-Ortiz R., Tomás-Vidal A., Gallardo-Álvarez F. J. (2018). Partial and Total Replacement of Fishmeal by a Blend of Animal and Plant Proteins in Diets for *Seriola dumerili*: Effects on Performance and Nutrient Efficiency. *Aquaculture Nutrition*.

[68] Cabral E. M., Fernandes T. J. R., Campos S. D. (2013). Replacement of Fish Meal by Plant Protein Sources up to 75% Induces Good Growth Performance Without Affecting Flesh Quality in Ongrowing Senegalese Sole. *Aquaculture*.

